# Editorial: The ever-changing scenario of first line treatment of metastatic renal cell carcinoma

**DOI:** 10.3389/fonc.2023.1183749

**Published:** 2023-03-20

**Authors:** Maria Giuseppa Vitale, Marco Maruzzo, Roberto Sabbatini

**Affiliations:** ^1^ Medical Oncology, Department of Oncology and Haematology, Azienda Ospedaliero-Universitaria di Modena, Modena, Italy; ^2^ Medical Oncology Unit 1, Department of Oncology, Istituto Oncologico Veneto IOV-Istituto di Ricovero e Cura a Carattere Scientifico (IRCCS), Padua, Italy

**Keywords:** renal cancer, first line treatment, combination therapy, prognosis, nephrectomy

Renal cell carcinoma (RCC) accounts for 4.1% of all new cancers, with a median age at diagnosis of 64 years. Approximately 85% of kidney tumors are RCC (renal cell carcinoma), and 70% of these have a clear cell histology (ccRCC) (1–4).

For selected patients with limited disease, partial nephrectomy (PN) is an option to treat RCC with oncological outcome comparable to radical nephrectomy. In their meta-analysis, Bai et al. investigated the effect of positive surgical margins (PSM) in term of prognosis. The pooled results showed a significant impact of PSM during PN in term of risk of recurrence (odd ratio, OR 3.93) and risk of metastasis (OR 4.63). However, PSM did not affect the risk of all-causes or cancer-specific death. Therefore, the study supports close monitoring for patients with PSM after PN.

From a surgical perspective, tumor thrombus (TT) is often associated to primary RCC. In a mono-institutional cohort, Wang et al. analysed the impact in survival of different histologies (clear and non-clear cell) in RCC with TT. The presence of thrombosis was more common in papillary RCC (pRCC); the oncological outcomes (both progression-free and overall survival) were worse for this subgroup of patients. Moreover, histology is independently associated to worst OS and PFS and strict follow-up should be followed for pRCC patients with TT.

Tyrosine kinase inhibitors (TKIs) such as Sunitinib or Pazopanib have been widely used in first-line treatment. Immunotherapy has provided a revolution in treatment options and recently, combination therapy ICI (Immune checkpoint inhibitors) -TKI and ICI-ICI have demonstrated remarkable efficacy in patients with mRCC. Tumour histology and risk stratification of patients is important in therapy selection.

In the paper by Dong et al., the authors investigated the expression profile of TFE3 of in 796 patients with RCC and the clinicopathological features as well as prognosis of TFE3-positive RCC. 91 (91/796, 11.4%) patients were TFE3 positivity expression and only 31 (31/91, 34.1%) of the patients were diagnosed with Xp11.2 translocation RCC. In this study, nuclear TFE3 expression is not specific to the Xp11.2 translocation RCC. Moreover, the positive TFE3 expression is associated with tumour progression and poor prognosis in patients with RCC irrespective of the presence of TFE3 translocation.

In the paper by Qin et al., they conduced a Bayesian network meta-regression analysis to provide a head-to-head comparison of first line therapeutic immune checkpoint inhibitors (ICI) and tyrosine kinase inhibitor (TKI) combinations for metastatic renal cell carcinoma. After analysing a total of 22 randomised clinical trials (RCTS) the combination Lenvatinib-Pembrolizumab shows dominance of progression free survival (PFS) and Pembrolizumab-Axitinib shows superiority in overall survival (OS). After meta-regression analysis, for hazard ratio (HR) of PFS, Lenvatinib-Pembrolizumab shows advantages; for HRs of OS, Pembrolizumab-Axitinib demonstrates superiority; for objective response rate (ORR), Lenvatinib-Pembrolizumab provides better results. About tolerability, the combination Atezolizumab-Bevacizumab is better. The authors conclude that Pembrolizumab-Axitinib should be recommended as the optimal therapy for the first line treatment of mRCC thanks to the lower toxicity and the higher quality of life.

Although the adverse events of both drug classes of combination therapy are now well-known, it may be difficult to recognise which drug is related to a particular adverse event. Boutros et al. reported two cases of patients with muscle enzyme elevation in association with hypothyroidism during treatment with Pembrolizumab-Axitinib for mRCC. The myopathy rapidly resolved after hormone replacement therapy with levothyroxine. This adverse event is rare and in the differential diagnosis with immune-related myositis which has different pathogenesis and course.

In a changing scenario with new multiple combinations of drugs effective for RCC, other mechanisms of action are desirable. Wang et al. the role of Glutamate dehydrogenase 1 (GLUD1), which plays a critical role in the malignancy of diverse tumors. They found that GLUD1 has a novel tumor-suppressing role of GLUD1 in ccRCC, different from that in other tumors, and these results provide a theoretical basis for GLUD1 as a therapeutic target and prognostic marker in renal cancer.

In summary, in the ever-changing scenario of first line treatment for RCC, this collection highlights the significance of appropriate surgery and pathological reports, to ensure adequate follow-up to patients with a greater risk of relapse. Moreover, it is of crucial significance the appropriate choice of the first line therapy among all the different approved treatments, to maximise patients’ benefit. Finally, identifying biomarkers or possible targets for new drugs development is critical to find out treatment strategies for RCC patients.

## Author contributions

MV, MM, RS drafted, reviewed, edited, finalized the manuscript. All authors contributed to the article and approved the submitted version.
